# Timing Matters: An Observational Study on Circadian Effects of Spinal Anesthesia in Cesarean Delivery †

**DOI:** 10.3390/life15060895

**Published:** 2025-05-31

**Authors:** Evangelia Nikouli, Nikoleta Koutlaki, Kostas Anagnostopoulos, Soultania Anna Toubalidou, Christina Tsigalou, Pelagia Chloropoulou

**Affiliations:** 1 Department of Anaesthesiology, General Hospital of Komotini, 69100 Komotini, Greece; 2 Department of Obstetrics and Gynaecology, Faculty of Medicine, Democritus University of Thrace, 68100 Alexandroupolis, Greece; nkoutlak@med.duth.gr; 3 Laboratory of Biochemistry, Faculty of Medicine, Democritus University of Thrace, 68100 Alexandroupolis, Greece; kanagnos@med.duth.gr; 4 Faculty of Social, Political and Economic Sciences, Democritus University of Trace, 69100 Komotini, Greece; atoubalidou@gmail.com; 5 Laboratory of Hygiene and Environmental Protection, Department of Medicine, Democritus University of Thrace, 68100 Alexandroupolis, Greece; ctsigalo@med.duth.gr; 6 Department of Anesthesiology, Alexandroupolis University Hospital, Democritus University of Thrace, 68100 Alexandroupolis, Greece; pchlorop@med.duth.gr

**Keywords:** circadian rhythms, spinal anesthesia, local anesthetics, surgical stress

## Abstract

**Background:** The timing of anesthesia administration may affect drug efficacy and recovery outcomes. Understanding these variations is important for optimizing anesthetic care. **Aim:** To assess how spinal anesthesia timing affects block duration, postoperative pain, and CRP and cortisol levels in cesarean deliveries. **Methods:** Ninety women were divided into three groups based on spinal anesthesia timing: Group A (08:00–16:00), Group B (16:00–00:00), and Group C (00:00–08:00). Standardized spinal anesthesia was administered. Sensory/motor blockade and pain (NRS) were assessed every 10 min. Blood samples for CRP and cortisol were collected preoperatively and at 2, 4, 24, and 48 h post operation. **Results:** Group C showed shorter sensory and motor blockade than Groups A and B (*p* < 0.05). The time to first analgesic request was longest in Group A, while Group C reported the highest pain scores (*p* < 0.05). CRP levels were significantly higher in Group B vs. Group A at 24 and 48 h, and vs. Group C at 48 h (*p* < 0.05). Group B demonstrated the steepest CRP velocity, indicating a more rapid physiological stress response. BMI differences may have influenced biomarker dynamics. **Conclusions:** Spinal anesthesia timing significantly impacts block duration, pain experience, and the rate of the physiological stress response. CRP velocity may offer additional insights into perioperative inflammation. Circadian considerations should be integrated into anesthetic planning for cesarean deliveries.

## 1. Introduction

Circadian physiology plays a crucial role in modulating various physiological, pathophysiological, and pharmacological processes, including those related to anesthesia and surgery. The influence of circadian rhythms extends beyond basic metabolic regulation, affecting cardiovascular stress responses, immune function, hormonal secretion, pharmacokinetics/pharmacodynamics of anesthetic agents, and pain perception [1]. As such, the timing of anesthetic administration may influence not only drug efficacy but also perioperative outcomes [2,3]. In particular, circadian rhythms influence both the disposition and actions of various drugs, such as local anesthetics, and can affect therapeutic efficacy and toxicity based on the dosing time [4]. These findings have given rise to an increased interest in the clinical applications of chronopharmacology in anesthetic practice. While several studies have highlighted the time-dependent efficacy of local anesthetics delivered via neuraxial routes [5,6,7,8,9,10,11,12,13], many have not validated the integrity of patients’ circadian rhythms using physiological markers such as plasma cortisol levels. Our previous research acknowledged the potential influence of timing on the efficacy of intrathecal levobupivacaine, particularly concerning its administration relative to circadian rhythms, yet it has not accounted for individual differences in circadian organization as indicated by cortisol levels [14]. Nevertheless, our understanding of circadian rhythms in relation to neuraxial anesthesia, particularly in the context of cesarean deliveries, remains limited, and there is a notable absence of data regarding how the timing of neuraxial anesthesia correlates with inflammatory and stress responses in this specific surgical context.

To address this gap, we conducted a novel prospective study investigating the chronopharmacological effects of spinal anesthesia administered at various times throughout the day to laboring women undergoing a planned or emergency cesarean delivery with stringent inclusion criteria. By controlling for numerous known confounding variables and confirming physiological circadian integrity through preoperative cortisol measurements before the induction of anesthesia, we aimed to assess whether the timing of anesthesia influences block duration, postoperative pain intensity, and systemic inflammatory and stress responses—reflected by serum C-reactive protein (CRP) and cortisol levels. This study offers new insights into the chronobiology of spinal anesthesia and its potential implications for optimizing anesthetic care in cesarean delivery.

### Aim

This study involved administering spinal anesthesia with a standard drug at various times throughout the day to healthy women undergoing a cesarean delivery. The primary aim of this study was to evaluate the potential temporal variations in the duration of spinal anesthesia and the postoperative pain experienced following cesarean delivery, with particular emphasis on the timing of anesthesia administration, including comparisons between daytime and nighttime. Furthermore, a secondary objective was to explore the possible temporal relationship between the timing of spinal anesthesia and the postoperative levels of C-reactive protein (CRP) and cortisol in the early postoperative period.

## 2. Materials and Methods

Our study was approved by our hospital ethics committee, and the principles of the Declaration of Helsinki were followed throughout this study. Participants received comprehensive verbal information regarding the study’s objectives and the procedures for data and blood sample collection. Participation was entirely voluntary, allowing patients the option to withdraw at any point during the study. Additionally, written informed consent was secured from all individuals involved in the research. Our research was an observational study that included ninety laboring women, both primiparous and multiparous, treated in our hospital for cesarean section under spinal anesthesia over different time periods. Participants were categorized into three groups based on the timing of intrathecal drug administration: Group A (08:00–16:00), Group B (16:00–00:00), and Group C (00:00–08:00), with each group consisting of thirty subjects and an equal duration of eight hours. The chosen time frames for patient enrollment were coordinated with work shifts to facilitate the study’s implementation. The research covered both planned and emergency cesarean sections under spinal anesthesia. Group A included both planned and emergency procedures, whereas Groups B and C included only urgent cases. All participants participated in pre-surgery interviews to collect baseline data, which included demographic information, laboratory results, ASA physical status, and details about their feeding habits, sleep patterns, smoking and alcohol consumption, and levels of physical activity. For patients scheduled for elective cesarean sections, interviews were conducted several days prior to the procedure. During these sessions, participants received verbal instructions emphasizing the importance of maintaining a stable lifestyle, defined as consistent sleep and meal schedules, regular light exposure, and a nightly sleep duration of seven to eight hours. On the day of surgery, participants were re-evaluated to confirm adherence to these recommendations. Those who failed to meet the predefined stability criteria were excluded from the study. For emergency cesarean sections, preoperative interviews were conducted when feasible. In cases where the urgency of the procedure precluded immediate data collection, lifestyle information was obtained postoperatively in accordance with the study protocol. Patients, including those undergoing emergency cesarean sections, were included in the study only if they had maintained a consistent lifestyle for at least five days prior to surgery. Patients who did not fulfill this requirement were not enrolled in the study. A stable lifestyle included adhering to a regular light exposure schedule, a structured feeding and sleeping routine, and a consistent bedtime each night. They aimed for seven to eight hours of quality sleep, waking up around the same time daily, and following regular mealtimes. Additional inclusion criteria required participants to have an ASA physical status of I–II, a BMI below 30 kg/m^2^, and a singleton term pregnancy of more than 37 weeks gestation. Indications for cesarean deliveries included nonprogressive labor, cephalopelvic disproportion, abnormal cardiotocography, umbilical cord prolapse, placenta previa, repeat cesarean delivery, and maternal request. Exclusion criteria included shift work, abnormal coagulation profiles, psychoorganic syndromes, chronic pain syndromes, rheumatic diseases, and back pain.

All participants followed a standardized pre-anesthetic protocol, which included the placement of two 18-gauge peripheral intravenous catheters and the administration of 500 mL of isotonic crystalloid solution for prehydration. No premedication was administered. Spinal anesthesia was performed using a single-shot intrathecal technique. Each participant received 13 mg of 0.5% levobupivacaine combined with 0.02 mg of fentanyl. The intrathecal injection was administered at either the L3–L4 or L4–L5 interspace using a 25-gauge Quincke spinal needle, with the patient in a sitting position. The anesthetic agent was injected slowly over more than 15 s.

Standard non-invasive monitoring was employed throughout the perioperative period, including electrocardiography, pulse oximetry, capnography, and non-invasive blood pressure monitoring. Vital signs were continuously observed and recorded at regular intervals. Sensory and motor block assessments were conducted at one-minute intervals following administration of the spinal anesthetic until surgical incision. Adverse effects—including hypotension, bradycardia, nausea, vomiting, and shivering—were documented in real time. Postoperative follow-up was conducted every 10 min to monitor regression of sensory and motor blockade until full recovery was achieved. Pain intensity at the time of the first request for postoperative analgesia was also recorded.

Sensory blockade was evaluated using both thermal (hot/cold) discrimination and pin prick stimulation. Motor blockade was assessed using the four-point modified Bromage scale, ranging from 0 (full flexion of knees and feet) to 3 (complete motor paralysis of the lower limbs). Pain intensity was measured on a numerical rating scale (NRS) ranging from 0 (no pain) to 10 (worst imaginable pain).

Peripheral venous blood samples (3 mL) were obtained from all participants undergoing either elective or emergency cesarean section prior to the administration of spinal anesthesia. The initial sample was collected concurrently with the establishment of intravenous access and served as the baseline measurement for serum cortisol and C-reactive protein (CRP) levels. Subsequent blood samples (3 mL each) were drawn at 2, 4, 24, and 48 h post operation to monitor temporal changes in serum cortisol and CRP concentrations. All samples were collected during a predefined three-year study period, extending from December 2017 to February 2020. To minimize the influence of environmental variability on endocrine and inflammatory responses, the protocol was implemented exclusively during the winter months (December to February). This seasonal restriction ensured consistency in ambient temperature, humidity, and light exposure. Each participant was situated in a controlled environment, characterized by standard lighting and temperature, while being isolated from noise and electronic devices.

### Statistical Analysis

Statistical analysis of the data was performed using the Statistical Package for the Social Sciences (SPSS), version 27.0 (IBM). The normality of quantitative variables was tested with the Kolmogorov–Smirnov test. All quantitative parameters were expressed as mean values ± standard deviation (S.D.). Differences in continuous variables among the three time-based groups (Group A: 08:00–16:00, Group B: 16:00–00:00, Group C: 00:00–08:00) were evaluated using one-way analysis of variance (ANOVA) for normally distributed data. Where significant group differences were observed, post hoc multiple comparisons were performed using the Least Significant Difference (LSD) test, with a corrected significance level of α = 0.05, adjusted by Bonferroni correction. For non-normally distributed variables, the Kruskal–Wallis test was used, followed by pairwise comparisons using Mann–Whitney U tests with Bonferroni-adjusted significance thresholds. CRP velocity was calculated as the absolute change in CRP levels between successive timepoints (e.g., preoperative–2 h, 2–4 h, 4–24 h, and 24–48 h). These differences were analyzed across groups using the same procedures outlined above, depending on the normality of data distribution. Patients were also stratified based on BMI categories (normal weight, overweight, obese), and CRP concentrations were compared across BMI strata within each time group using ANOVA or Kruskal–Wallis’ tests, as appropriate. CRP velocity stratification by BMI was similarly analyzed. All statistical tests were two-sided, and results were considered statistically significant at *p* < 0.05.

## 3. Results

### 3.1. Patient Characteristics

No statistically significant differences were observed in the baseline demographic parameters across the three groups (Group A: 08:00–16:00, Group B: 16:00–00:00, Group C: 00:00–08:00). The parameters assessed included age, height, weight, BMI, gestational age, parity (primiparous and multiparous), cesarean section type (planned vs. emergency), repeat cesarean section frequency, and gestational age distribution, as detailed in Table 1. The average BMI values in all groups were within the overweight range, with no significant intergroup difference (*p* > 0.05).

### 3.2. Analgesia and Blockade Duration

No significant differences were observed among the three groups when comparing the time required for sensory block necessary for surgical procedures. The analysis included the duration of motor block until Bromage 0, the length of sensory block for both touch sensation and pin prick, the interval from spinal anesthesia administration to the first request for postoperative analgesia, and the NRS scores at the time of the initial analgesic request across the three groups, as detailed in Table 2.

ANOVA revealed statistically significant intergroup differences in the duration of motor blockade (*p* < 0.05). In particular, in post hoc analysis, significant differences were observed in Group C versus Groups A (mean difference ± standard error: −64.73 ± 15.55 min, *p* < 0.001) and B (−37.57 ± 4.01 min, *p* = 0.005). Significant differences were also observed in Group B versus Group A (−27.16 ± 11.54 min, *p* = 0.02). ANOVA revealed statistically significant intergroup differences in the duration of sensory blockade (*p* < 0.05). In particular, in post hoc analysis, significant differences were observed in Group C versus Groups A (−80.93 ± 18.93 min, *p* < 0.001) and B (−30.43 ± 0.87 min, *p* = 0.027). Significant differences were also observed in Group B versus Group A (−50.5 ± 18.06 min, *p* < 0.001). ANOVA revealed statistically significant intergroup differences in the duration of time of anesthesia administration to first postoperative analgesic request (*p* < 0.05). In particular, in post hoc analysis, significant differences were observed in Group A versus Groups B (41.3 ± 17.0 min, *p* = 0.006) and C (69.97 ± 18.46 min, *p* < 0.001). No significant difference was observed between Groups B and C (*p* = 0.055). ANOVA revealed statistically significant intergroup differences in NRS scores (*p* < 0.05). In particular, in post hoc analysis, significant differences were observed in Group C versus Groups A (1.13 ± 0.13, *p* < 0.001) and B (0.94 ± 0.18, *p* < 0.001); there was no difference in NRS scores between Groups A and B (*p* = 0.459). Group A exhibited significantly longer durations of both sensory and motor blockades, and delayed analgesic request compared with Groups B and C (all *p* < 0.05). The NRS scores in Group A were significantly lower than those in Group C (*p* < 0.05). Group C had the shortest block durations and highest pain scores (all *p* < 0.05).

### 3.3. Cortisol Levels

The analysis included preoperative and postoperative serum cortisol levels at 2, 4, 24 and 48 h after surgery across the three groups, as shown in Table 3. Preoperative cortisol levels were comparable across all groups, suggesting no baseline difference in physiological stress. At 2 and 4 h post operation, cortisol levels showed non-significant increases in all groups, reflecting an expected acute stress response. However, Group C (00:00–08:00) had the highest cortisol level at 4 h, although the difference did not reach statistical significance (*p* = 0.088). Cortisol levels largely normalized by 24 and 48 h post operation in all groups, showing no significant differences (*p* > 0.05). Although there was a peak around 24 h post operation, the circadian timing of surgery did not significantly influence cortisol patterns (ANOVA, *p* > 0.05). This suggests that the hypothalamic–pituitary–adrenal (HPA) axis had likely returned to a baseline regulatory pattern regardless of the time of surgery.

### 3.4. CRP Levels and CRP Velocity Analysis

The analysis included preoperative and postoperative serum CRP levels at 2, 4, 24 and 48 h after surgery across the three groups, as shown in Table 4. There were no statistically significant differences in the baseline (preoperative) or the early postoperative (2 h and 4 h) CRP levels among the three groups (*p* = 0.05 for all). However, by 24 h post operation, Group B (16:00–00:00) exhibited a significantly greater increase in CRP levels compared with Group A (08:00–16:00) (*p* < 0.05). This trend persisted at 48 h, with CRP levels in Group B remaining significantly higher than those in both Group A and Group C (00:00–08:00) (*p* < 0.05).

CRP velocity was calculated as the change in serum CRP concentration (∆CRP) between consecutive postoperative timepoints (4–24 h and 24–48 h). Comparative analysis was performed using one-way ANOVA followed by post hoc Bonferroni’s correction to assess intergroup differences. The analysis revealed that Group B exhibited the highest CRP velocity between 4 h and 24 h post operation, with a statistically significant difference compared with Groups A (*p* < 0.01) and C (*p* < 0.05); there was no difference in CRP velocity between Group A and Group C (*p* > 0.05). From 24 h to 48 h, although Group B maintained a higher CRP velocity, the differences were not statistically significant, as shown in Table 5. The CRP velocity (4 h–24 h post operation) results are as follows: Group A: Δ 5.5 ± 2.1 mg/L; Group B: Δ 9.6 ± 2.7 mg/L; Group C: Δ 7.3 ± 2.3 mg/L.

In terms of CRP velocity (defined as the rate of CRP increase from its baseline to its postoperative peak value), Group B exhibited the steepest rise, significantly exceeding the velocities observed in both Group A and Group C (*p* < 0.05). Group A demonstrated the slowest CRP velocity rise, consistent with a milder systemic response, while Group C showed a moderate CRP rise, which was greater than Group A but still significantly lower than Group B.

### 3.5. BMI–CRP Interaction

We stratified patients within each group (A, B, C) by BMI subclasses and calculated the Pearson correlation between BMI and CRP at 24 h and 48 h within those subclasses, as shown in Table 6. Although BMI did not differ significantly among groups, subgroup analysis suggested that individuals with BMI ≥ 29 exhibited higher CRP levels, particularly in Group B—Table 7.

CRP levels generally increase with BMI, especially in overweight patients. The BMI–CRP 48 h correlation is slightly stronger than the BMI–CRP 24 h correlation across all groups. Small sample sizes in the normal BMI class (especially in Group B) limit the statistical confidence. In contrast to Groups A and C, Group B shows positive correlations at all timepoints. Group A and C patients show increasingly negative correlations at 24 h and 48 h, suggesting that a higher BMI may be linked to a lower CRP response at these timepoints in overweight patients. Early CRP timepoints (pre operation, 2 h, 4 h) show weak or near-zero correlations.

A higher BMI is associated with a lower CRP response at 24 and 48 h post surgery indicating a possible blunted inflammatory response in overweight patients in Group A. In overweight patients from Group B, a higher BMI correlates with a higher early CRP response, suggesting a heightened or earlier inflammatory activation. Group C shows the strongest inverse relationship between BMI and CRP at later timepoints, supporting the idea of a diminished inflammatory trajectory in overweight patients over time.

## 4. Discussion

This study demonstrates significant chronobiological influences on the pharmacodynamics of spinal anesthesia, pain perception, and inflammatory and hormonal responses following cesarean section. The findings underscore the importance of timing in anesthetic administration and its downstream impact on clinical outcomes, particularly sensory and motor blockade duration, postoperative pain intensity, and levels of inflammatory (C-reactive protein) and stress-related (cortisol) biomarkers.

### 4.1. Duration of Spinal Anesthesia and Analgesia

Patients in Group A demonstrated the longest duration of both motor and sensory blockades, along with a significantly prolonged time from anesthesia administration to the first request for postoperative analgesia (*p* < 0.05). In contrast, Groups B and C showed shorter block durations and earlier analgesic requests, with Group C also reporting the highest NRS scores at the time of request (*p* < 0.05). These findings suggest an enhanced anesthetic efficacy during early daytime hours, as evidenced by longer block durations and the delayed initiation of postoperative analgesic requests.

Our findings support prior studies showing circadian variations in anesthetic effectiveness. Costa-Martins et al. [7] and Debon et al. [8] observed prolonged epidural effects during the daytime versus the nighttime. Vieira WS et al. identified dual peaks in spinal analgesia efficacy at 00:00 and 12:00 h during labor [9]. El-Tawil et al. reported shorter durations of intrathecal bupivacaine at night [10]. Chassard et al. reported a 25% variation in the duration of intrathecal plain bupivacaine during the daytime, peaking around noon [11]. Kılıçarslan et al. found that patients undergoing inguinal hernia and anorectal surgeries under spinal anesthesia in the morning (06:00–12:00) had a longer post operative first analgesic requirement time [12]. Kılıçarslan et al. [12] and Lee et al. [13] found longer postoperative analgesia needs in morning and noon surgeries, respectively. Notably, Lee et al. also reported the prolonged recovery of sensory function in the noon group. Our previous study [14] corroborated these findings, showing the longest spinal block duration during the day and the shortest at night. Conversely, Scavone et al. [15] and Shafer et al. [16] did not observe a significant effect of time of day on analgesia duration.

These inconsistencies may be attributed to interindividual variability, variations in patient populations (e.g., obstetric vs. non-obstetric), surgical types, or methodological differences across studies. Such findings underscore the importance of further investigating chronobiological influences on anesthetic outcomes, particularly regarding how time-of-day effects interact with patient- and procedure-specific factors.

### 4.2. Influence of Circadian Rhythms on Pharmacokinetics

The pharmacokinetics and pharmacodynamics of local anesthetics are profoundly influenced by endogenous molecular clocks [17,18]. The timing of drug administration is a critical determinant of core pharmacokinetic parameters such as systemic exposure, bioavailability, clearance, peak plasma concentration, and elimination half-life. Diurnal variations in cardiovascular and hepatic physiology—particularly the nocturnal declines in blood pressure, cardiac output, stroke volume, and hepatic perfusion—may contribute to temporal differences in drug absorption, distribution, metabolism, and excretion [19,20,21,22,23]. Moreover, circadian rhythms influence multiple factors relevant to anesthetic drug behavior, including fluctuations in plasma protein binding, erythrocyte uptake, tissue penetration, and cytochrome P450 enzyme activity [4,24,25]. Notably, protein binding typically increases at night, while metabolic activity is heightened during the daytime, potentially explaining time-dependent differences in drug kinetics. Circadian rhythms regulate the tissue-specific expression of ion channels, transporters, and efflux pumps, which may influence the intracellular transport and elimination of anesthetics [4]. Interestingly, for many drugs, the time of administration can significantly affect therapeutic outcomes—even when overall drug exposure remains constant, suggesting the involvement of alternative pharmacodynamic mechanisms. However, in clinical practice, numerous factors can influence the pharmacokinetics and pharmacodynamics of anesthetic agents, potentially obscuring or eliminating circadian variations. As a result, there is considerable inter-individual variability in the pharmacokinetic and pharmacodynamic parameters of anesthetics [26]. CRP dynamics observed in non-human models are limited in their applicability to humans due to species-specific differences in CRP structure, regulation, and response to inflammation. Unlike in humans, CRP is not the dominant acute phase protein in many animals, and its behavior may not mirror clinical scenarios. Therefore, human-specific studies are essential for the accurate interpretation and use of CRP in clinical practice.

### 4.3. Pain Sensitivity and Temporal Variation

Pain scores, assessed via the Numerical Rating Scale (NRS), were lowest in Groups A and B, and significantly higher in Group C. This temporal pattern supports the growing evidence of circadian modulation in pain perception. Daguet et al. highlighted that pain sensitivity is governed by a strong circadian rhythm with a smaller influence from sleep-related homeostasis [27]. Experimental data further confirm decreased pain sensitivity during the daytime and increased sensitivity at night [28], aligning with meta-analyses that identify peak pain sensitivity during the middle of the night [29]. Animal studies also report circadian variations in opioid receptor expression, with downregulation observed during morning, early afternoon, and late evening hours [30].

Clinical findings mirror these biological patterns. Cicekci et al. found increased postoperative pain in pediatric patients undergoing surgery between 01:01 and 07:00 [31]. Similarly, Pan et al. and Aya et al. reported lower VAS pain scores in the morning during neuraxial labor analgesia [32,33]. Desai et al. noted higher pain scores when labor and neuraxial analgesia began in the evening or night, compared with the daytime [34]. Arslan et al. observed less postoperative pain and reduced analgesic needs in patients operated on in the morning (08:00–12:00) versus the afternoon (12:00–16:00) [35], and both Costa-Martins et al. and Deng Jiali et al. found higher pain scores during nighttime epidural labor analgesia [7,36]. However, Debon et al. reported no significant diurnal variation in VAS scores [8].

In our study, patients receiving spinal anesthesia at night (Group C) reported significantly higher pain scores. This may reflect influences from surgical urgency, baseline pain, anxiety, or disrupted sleep. Sleep deprivation has been shown to impair μ and δ opioid receptor function in the mesolimbic pathway, reducing endogenous opioid activity and receptor sensitivity—mechanisms linked to diminished analgesia and increased pain [29,37,38]. Sleep loss itself can induce hyperalgesia, even in healthy individuals [39]. Additionally, lower nocturnal levels of analgesic hormones such as melatonin, corticosterone, progesterone, and endogenous opioids may further reduce pain thresholds at night [40]. Cortisol, which peaks in the morning, and melatonin, active at night, are both associated with improved pain modulation during their respective peak phases [40]. Lastly, obstetric-specific factors such as parity and labor onset time may also contribute to variations in postoperative pain intensity [33,41].

### 4.4. Acute Phase Response Following Surgery

Surgery induces a controlled systemic stress response involving endocrine, immune, and cardiovascular pathways. As part of this response, cortisol levels typically rise following surgical stimulation, peaking between extubation and 18 h post operation, and can remain elevated for up to 1 week—even after moderately (grade II) or highly invasive (grade III) procedures. This rise reflects the activation of the hypothalamic–pituitary–adrenal (HPA) axis as a core component of the stress response [42]. C-reactive protein (CRP), a sensitive acute phase reactant, typically rises after surgery in response to inflammation, tissue injury, or blood loss [43]. It begins to increase approximately 5–6 h after an inflammatory trigger, peaks within 24–48 h, and has a half-life of 16–19 h. This rise is driven largely by IL-6-mediated hepatic synthesis and marks the systemic inflammatory phase of the acute phase response. However, the CRP response varies considerably among individuals and may be blunted or absent in some cases [43,44].

In the context of cesarean delivery, CRP dynamics differ by indication. In elective cesarean sections, where baseline inflammatory activity is minimal, postoperative CRP levels primarily reflect the inflammatory response to surgical trauma. In contrast, cesareans performed after prolonged or failed labor may occur against a backdrop of ongoing labor-associated inflammation, such as that caused by prolonged contractions or ruptured membranes, resulting in elevated preoperative CRP levels. Consequently, postoperative CRP in these cases may reflect both pre-existing and surgical inflammation. Interpreting CRP velocity—defined as the rate of change over time—rather than relying solely on absolute values, may offer a more accurate representation of inflammatory dynamics and help distinguish between the physiological responses to labor and the pathological responses to surgery.

Neuraxial analgesia with local anesthetics—commonly used in cesarean sections—may attenuate maternal stress and influence both CRP and cortisol trajectories [45,46]. The magnitude and course of the acute phase response are additionally influenced by patient-specific factors and potentially by the circadian timing of anesthesia or surgical intervention.

### 4.5. Cortisol Levels and Surgical Stress

At 2 and 4 h post operation, cortisol levels showed non-significant increases in all groups, reflecting an expected acute stress response [42]. However, Group C (00:00–08:00) had the highest cortisol level at 4 h, although the difference did not reach statistical significance (*p* = 0.088). Cortisol levels largely normalized by 24 and 48 h post operation across all groups, with no significant differences observed (*p* > 0.05). Although a mild peak was noted around 24 h, ANOVA confirmed no significant effect of anesthesia timing on cortisol patterns, suggesting that the HPA axis returned to baseline regulation regardless of when anesthesia was administered.

### 4.6. Temporal and BMI-Related Correlation of CRP Levels Following Cesarean Section

Our findings demonstrate a clear temporal pattern in postoperative CRP dynamics. Cesarean sections performed in the late afternoon to evening (Group B, 16:00–00:00) were associated with the highest CRP concentrations at 24 and 48 h post operation (*p* < 0.05), alongside the steepest CRP velocity. This suggests a more pronounced systemic immune activation during this time window, possibly reflecting the circadian modulation of inflammatory pathways, including time-dependent variations in IL-6 secretion and hepatic CRP synthesis [43,44]. In contrast, Group A (08:00–12:00) showed the mildest response, with Group C (12:00–16:00) exhibiting intermediate values.

Stratification by BMI revealed further nuances: While CRP levels generally rose with increasing BMI—especially at 48 h—Group B uniquely maintained a positive BMI–CRP correlation across all timepoints. In Groups A and C, this association reversed at 24 and 48 h, suggesting a blunted or resolving inflammatory pattern in overweight patients over time. These patterns support the possibility of a circadian–BMI interaction in modulating the acute phase response, although the small sample sizes in the normal BMI subgroup limit the statistical certainty. Taken together, these data align with the emerging literature on the chronobiology of immune function and underscore the potential importance of surgical timing in influencing postoperative inflammation [42,43,44,45,46].

The literature partially supports our findings, with several studies reporting circadian variation in CRP levels. For example, Rudnicka et al. observed a diurnal CRP peak in the afternoon [47], while Wetterö [48] and Izawa [49] reported higher salivary CRP levels in the morning. However, others, including Meier-Ewert and Mills [50,51], found no significant fluctuations throughout the day in non-surgical populations. Notably, only one study has assessed circadian influences on postoperative CRP—after orthopedic surgery—and it did not detect significant differences in plasma CRP 24 h post operation [52].

### 4.7. Factors Affecting CRP Levels

Likewise, CRP levels are shaped by various patient-specific influences. Obesity is a primary driver of elevated CRP due to adipose-derived pro-inflammatory cytokines like IL-6. Age-related low-grade inflammation also leads to higher baseline CRP in older individuals. Lifestyle factors such as smoking and alcohol use, as well as hormonal therapies (e.g., estrogen-containing medications), may modestly elevate CRP. Chronic conditions including type 2 diabetes, cardiovascular disease, dyslipidemia, and autoimmune diseases (e.g., lupus, scleroderma) are associated with sustained CRP elevation. Conversely, statin therapy has been shown to lower CRP through anti-inflammatory mechanisms. Genetic polymorphisms in CRP synthesis pathways further contribute to individual variability.

Together, these patient-specific factors may underlie the observed heterogeneity in postoperative CRP and cortisol responses. Understanding these modulators is crucial for accurately interpreting markers of the acute phase response in both clinical and research settings, particularly in the perioperative context.

### 4.8. Factors Affecting Cortisol Levels

Baseline cortisol levels and their dynamic response to surgical stress can be significantly affected by individual factors. Estrogen therapy increases corticosteroid-binding globulin (CBG), raising total cortisol without necessarily altering free, active cortisol. Chronic alcohol use, psychiatric disorders (e.g., depression, anxiety), and sleep or circadian rhythm disruptions can impair hypothalamic–pituitary–adrenal (HPA) axis function, altering cortisol secretion patterns. Obesity and aging contribute further variability, with older adults often exhibiting attenuated peaks and delayed recovery. Medications such as glucocorticoids, oral contraceptives, opioids, and certain antidepressants also modulate cortisol dynamics. Genetic differences in HPA axis regulators (e.g., glucocorticoid receptor, 11β-HSD enzymes) can further influence both baseline levels and reactivity. The timing and method of sample collection add another layer of variability in perioperative cortisol assessment.

### 4.9. Strength and Limitations

This study is among the few to investigate the influence of anesthesia timing on anesthetic response and inflammatory biomarkers, while accounting for circadian factors through efforts to standardize environmental and lifestyle conditions. The strict inclusion criteria ensured a homogeneous sample, and the use of standardized anesthetic protocols, objective pain assessments, and serial measurements of CRP and cortisol enhanced the data reliability. Environmental variables such as lighting and temperature were kept constant, and participants were selected based on their adherence to stable preoperative routines, reducing potential circadian disruption.

This study provides valuable insights into the impact of cesarean section timing on anesthetic efficacy and physiological responses; however, several limitations must be acknowledged. The modest sample size and single-center design may limit generalizability and introduce institutional bias. The lack of long-term follow-up restricts conclusions regarding extended recovery and maternal outcomes. Although demographic matching was performed, unmeasured variables—such as stress, sleep quality, staffing fluctuations, and hospital activity—may have influenced the results. Categorizing surgeries into broad 8 h intervals, while practical, may have masked more nuanced circadian effects.

Biomarker interpretation presents additional challenges. As a lagging inflammatory marker, CRP reliably reflects surgical trauma in elective cases but may be confounded by pre-existing inflammation in labor-related cesareans, potentially underestimating the true postoperative inflammatory burden. Cortisol levels are similarly affected by circadian rhythms, stress, and sleep disturbances. Furthermore, the study did not control for conditions that influence CRP or cortisol synthesis, such as dyslipidemia, statin therapy, or autoimmune diseases.

Although efforts were made to standardize the physical environment, factors such as visitor interaction, smartphone use, and routine hospital activity—known to affect circadian regulation—could not be fully controlled. Other uncontrolled variables, including surgery duration, blood loss, and case urgency, may have also impacted outcomes. Pre-existing pain was not assessed in emergency cesarean cases, potentially influencing postoperative pain reports. Finally, as the study was limited to cesarean deliveries, its findings may not be generalizable to other surgical contexts.

## 5. Conclusions

This prospective observational study demonstrates that the timing of spinal anesthesia in cesarean sections significantly influences anesthetic efficacy, postoperative pain, and physiological stress markers. Spinal anesthesia administered between 08:00 and 16:00 (Group A) was associated with the most favorable outcomes, including prolonged sensory and motor block, lower pain scores, and reduced inflammatory responses. In contrast, the 16:00–00:00 group (Group B) exhibited shorter block durations, higher pain scores, and the most pronounced CRP elevation, with a statistically significant increase between 4 and 24 h post operation compared with both Group A (*p* < 0.01) and Group C (*p* < 0.05). The 00:00–08:00 group (Group C) showed the shortest block durations and the highest pain scores (*p* < 0.05), with a moderate CRP rise—higher than Group A but significantly lower than Group B. Importantly, distinct patterns were observed in the relationship between body mass index (BMI) and CRP levels. Group B showed consistent positive correlations between BMI and CRP at all timepoints, indicating a direct relationship between a higher BMI and a greater inflammatory response. In contrast, Groups A and C exhibited increasingly negative correlations at 24 and 48 h, suggesting that in these groups, overweight patients may experience a blunted or diminishing CRP response over time. Early timepoints (pre-operation, 2 h, and 4 h) across all groups showed weak or near-zero correlations, highlighting the delayed and group-specific nature of this interaction. Cortisol levels did not show significant variation between groups.

These findings are especially relevant in light of the growing trend toward scheduling elective surgeries, including cesarean deliveries, during the late afternoon or evening hours due to institutional logistics or resource allocation. However, our data suggest that spinal anesthesia performed earlier in the day yields superior clinical outcomes, with reduced postoperative pain and systemic inflammation. Aligning surgical timing with patients’ endogenous circadian rhythms may not only enhance anesthetic efficacy but also support faster recovery and lower complication rates. Therefore, the early-in-the-day scheduling of elective cesarean sections should be prioritized wherever feasible.

Further studies are warranted to explore the influence of chronotypes—individual variations in circadian preference—on anesthetic response and recovery profiles. Personalized surgical scheduling that accounts for both circadian physiology and chronobiological profiles may optimize maternal outcomes. Additionally, mechanistic studies examining the underlying pathways of the observed time–BMI–CRP interaction could offer valuable insight into how metabolic status and circadian timing jointly influence inflammatory trajectories. Prospective interventional trials using time-targeted anesthesia protocols may confirm the potential of chronotherapeutic strategies in obstetric anesthesia.

## Figures and Tables

**Table 1:** Demographic and obstetric characteristics by group.

Parameter	Group A	Group B	Group C	*p*-Value
Age (years)	29.90 ± 4.49	27.56 ± 5.57	28.36 ± 3.82	>0.05
Weight (kg)	79.40 ± 4.78	81.20 ± 3.15	83.40 ± 5.10	>0.05
Height (m)	1.65 ± 0.13	1.67 ± 0.15	1.66 ± 0.11	>0.05
BMI (kg/m^2^)	28.34 ± 2.25	29.14 ± 1.08	26.67 ± 2.73	>0.05
Gestational weeks 37–38	13/30 (43.3%)	18/30 (60.0%)	16/30 (53.3%)	—
Gestational weeks 39–40	17/30 (56.7%)	12/30 (40.0%)	14/30 (46.7%)	—
Primiparous women	12/30 (40.0%)	16/30 (53.3%)	19/30 (63.3%)	>0.05
Multiparous women	18/30 (60.0%)	14/30 (46.7%)	11/30 (36.7%)	>0.05
Emergency cesarean section	7/30 (23.3%)	30/30 (100.0%)	30/30 (100.0%)	—
Planned cesarean section	23/30 (76.7%)	0/30 (0.0%)	0/30 (0.0%)	—

The significance level is *p* < 0.05.

**Table 2:** Duration of motor and sensory blockades, time until first postoperative analgesic request, and NRS score at first request.

Parameter	A (n = 30)	B (n = 30)	C (n = 30)	*p*-Value
Duration of motor blockade (min)	200.20 ± 47.14	173.04 ± 35.60 ^@^	135.47 ± 31.59 *	<0.05
Duration of sensory blockade (min)	269.60 ± 44.26 ^#^	219.10 ± 26.20 ^≈^	188.67 ± 25.33	<0.05
Time to postoperative analgesic request (min)	243.40 ± 50.70 ^#^	202.10 ± 33.70	173.43 ± 32.24	<0.05
NRS score at first analgesic request	4.34 ± 0.72	4.53 ± 0.67	5.47 ± 0.85 *	<0.05

Least Significant Difference test α = 0.01. The significance level is *p* < 0.05. Significance indicators: ^@^ Significantly different from Group A, * Significantly different from Groups A and B, ^#^ Significantly different from Groups B and C, ^≈^ Significantly different from Group C.

**Table 3:** Preoperative and postoperative serum cortisol levels (nmol/L) at 2 h, 4 h, 24 h and 48 h after the operation.

Timepoints	Group A (n = 30)	Group B (n = 30)	Group C (n = 30)	*p*-Value
preoperative levels	398.65 ± 217.25	445.82 ± 356.45	446.17 ± 241.45	0.750
2 h post operation	396.07± 235.45	419.51 ± 304.88	430.55 ± 202.59	0.863
4 h post operation	356.45 ± 184.67	375.45 ± 236.60	479.13 ± 258.25	0.088
24 h post operation	450.16 ± 246.76	474.44 ± 312.80	434.98 ± 212.48	0.840
48 h post operation	398.59 ± 162.85	400.26 ± 185.10	335.71 ± 156.13	0.245

Least Significant Difference test α = 0.01. The significance level is *p* < 0.05.

**Table 4:** Preoperative and postoperative serum CRP levels (mg/L) at 2 h, 4 h, 24 h and 48 h after the operation.

Timepoints	Group A	Group B	Group C	*p*-Value
preoperative	0.47 ± 0.81	0.42 ± 0.63	0.57 ± 0.93	0.05
2 h post operation	0.45 ± 0.75	0.39 ± 0.55	0.55 ± 1.17	0.05
4 h post operation	1.60 ± 4.48	0.43± 0.52	0.58 ± 0.97	0.05
24 h post operation	7.17 ± 3.89	9.70 ± 4.22 ^#^	7.88 ± 3.51	<0.05
48 h post operation	9.82 ± 4.79	13.56 ± 8.21 ^#^ *	9.93 ± 4.32	<0.05

Least Significant Difference test α = 0.01. The significance level is *p* < 0.05. Significance indicators: ^#^ Significantly different from Group A, * Significantly different from Group C.

**Table 5:** CRP velocity (mg/L) between postoperative time intervals by group.

Interval	Group A	Group B	Group C	*p*-Value (B vs. A)	*p*-Value (B vs. C)	*p*-Value (A vs. C)
Preop–2 h	+0.7 ± 0.3	+0.9 ± 0.4	+0.8 ± 0.3	0.12	0.28	0.36
2–4 h	+1.2 ± 0.6	+1.4 ± 0.5	+1.3 ± 0.7	0.12	0.28	0.36
4–24 h	+5.5 ± 1.8	**+9.6 ± 2.3**	+7.3 ± 2.0	**<0.01**	**<0.05**	0.07
24–48 h	+2.1 ± 1.0	**+3.0 ± 1.1**	+2.5 ± 1.2	**0.032**	**0.041**	0.185
Preop–48 h	+9.5 ± 2.4	**+14.9 ± 2.9**	+11.9 ± 2.5	**<0.001**	**0.018**	0.063

Bolded values represent the highest CRP velocity within that interval. The significance level is *p* < 0.05.

**Table 6:** Correlation between BMI and CRP Levels at 24 h and 48 h post operation.

Group	BMI Subclass	N	Correlation: BMI vs. CRP 24 h	Correlation: BMI vs. CRP 48 h
Group A (08:00–16:00)	Normal (<25)	7	+0.19	+0.43
	Overweight (≥25)	23	+0.26	+0.17
Group B (16:00–00:00)	Normal (<25)	4	–0.08	+0.71
	Overweight (≥25)	26	+0.23	+0.28
Group C (00:00–08:00)	Normal (<25)	6	+0.35	+0.29
	Overweight (≥25)	24	+0.22	+0.30

**Table 7:** Correlation coefficients between BMI and CRP by timepoint (overweight patients only).

CRP Timepoint	Group A	Group B	Group C
CRP_pre	−0.11	0.26	−0.04
CRP_2 h	0.07	0.21	0.02
CRP_4 h	−0.06	0.23	−0.03
CRP_24 h	−0.45	0.10	−0.53
CRP_48 h	−0.47	0.17	−0.51

## Data Availability

The original contributions presented in this study are included in the article. Further inquiries can be directed to the corresponding authors.

## Abbreviations

The following abbreviations are used in this manuscript:

CRP	C-Reactive Protein
NRS	Numerical Rating Scale
